# Impact of Mycotoxins on Animals’ Oxidative Status

**DOI:** 10.3390/antiox10020214

**Published:** 2021-02-01

**Authors:** Alexandros Mavrommatis, Elisavet Giamouri, Savvina Tavrizelou, Maria Zacharioudaki, George Danezis, Panagiotis E. Simitzis, Evangelos Zoidis, Eleni Tsiplakou, Athanasios C. Pappas, Constantinos A. Georgiou, Kostas Feggeros

**Affiliations:** 1 Laboratory of Nutritional Physiology and Feeding, Department of Animal Science, Agricultural University of Athens, 11855 Athens, Greece; mavrommatis@aua.gr (A.M.); egiamouri@aua.gr (E.G.); stud217094@aua.gr (S.T.); stud210009@aua.gr (M.Z.); ezoidis@aua.gr (E.Z.); eltsiplakou@aua.gr (E.T.); cfeg@aua.gr (K.F.); 2 Chemistry Laboratory, Department of Food Science and Human Nutrition, Agricultural University of Athens, 11855 Athens, Greece; gdanezis@aua.gr (G.D.); cag@aua.gr (C.A.G.); 3 FoodOmics GR Research Infrastructure, Agricultural University of Athens, 11855 Athens, Greece; 4 Laboratory of Animal Breeding and Husbandry, Department of Animal Science, Agricultural University of Athens, 11855 Athens, Greece; pansimitzis@aua.gr

**Keywords:** aflatoxin, *Aspergillus*, mycotoxins, ochratoxin A, oxidative stress, poultry, ruminants, swine, zearalenone

## Abstract

Mycotoxins appear to be the “Achilles’ heel” of the agriculture sector inducing enormous economic losses and representing a severe risk to the health of humans and animals. Although novel determination protocols have been developed and legislation has been implemented within Europe, the side effects of mycotoxins on the homeostatic mechanisms of the animals have not been extensively considered. Feed mycotoxin contamination and the effects on the antioxidant status of livestock (poultry, swine, and ruminants) are presented. The findings support the idea that the antioxidant systems in both monogastrics and ruminants are challenged under the detrimental effect of mycotoxins by increasing the toxic lipid peroxidation by-product malondialdehyde (MDA) and inhibiting the activity of antioxidant defense mechanisms. The degree of oxidative stress is related to the duration of contamination, co-contamination, the synergetic effects, toxin levels, animal age, species, and productive stage. Since the damaging effects of MDA and other by-products derived by lipid peroxidation as well as reactive oxygen species have been extensively studied on human health, a more integrated monitoring mechanism (which will take into account the oxidative stability) is urgently required to be implemented in animal products.

## 1. Introduction

Mycotoxins are particularly toxic substances, which are mostly produced by three genera of fungi: *Aspergillus, Penicillium*, and *Fusarium* [1]. Mycotoxins have been characterized as secondary metabolites of low molecular weight [1,2]. They could be pathogenic or simply saprophytic and can show up on many food categories [2,3], either liquid or solid as well as on animal feed [1,2]. Contamination by mycotoxins can affect both human and animal health [1,2,3]. Some fungi can produce multiple mycotoxins [1]. In Table 1, the major fungi and produced mycotoxins are presented. The existence of mycotoxins has an outstanding repercussion on global economy and international commerce [1].

One of the most studied groups of mycotoxins is aflatoxins (AF), which are produced by the fungal genus *Aspergillus*, notably *A. flavus*, *A. parasiticus*, *A. ochraceus*, *A. carbonarius, A. niger* (lower significance), and seldomly by *A. pseudotamari* [3,4,5,6]. Favorable weather conditions are important for the production of aflatoxins, commonly apparent in tropical and sub-tropical countries [3]. Known compounds generated by *A. flavus* are aflatoxin B1 (AFB1), B2 (AFB2), G1 (AFG1), and G2 (AFG2) [2,3]. An in vivo metabolite found in animals fed AFB1 contaminated feed is M1 (AFM1) with known carry over to animal products like milk, eggs, and tissues [3]. Aflatoxins have been linked to human liver carcinogenesis and health issues in various animals that can lead to high mortality rates, especially in poultry [1,2,3].

Ochratoxins include several types, namely ochratoxin A, B, and C. Ochratoxin A (OTA), which can be found in several foods is the most profuse and damaging one. OTA are produced by fungal species belonging to *Aspergillus* and *Penicillium* genera, most notably *A. ochraceus, A. niger, A. carbonarius, A. westerdijkiae, A. glaucus, A. melleus, A. alliaceus, A. auricomus, P. verrucosum*, and *P. viridicatum* [1,5,6] (Table 1). Environmental conditions that favor OTA production are variable and depend on fungal genus [6]. Ochratoxins are toxic for the immune-, nervous system, and kidneys for both animals and humans [1,2,6]. Furthermore, they are responsible for DNA damage, cancer, and teratogens [2,6].

Zearalenone (ZEA) is produced mostly by the *Fusarium* genus. Specifically, ΖΕA is synthesized by the following fungal species: *F. graminearum*, *F. roseum*, *F. culmorum*, *F. equiseti*, *F. cerealis*, *F. verticillioides*, *F. incarnatum*, *F. crookwellense*, and *F. semitectum* [2] (Table 1). The great interest in their study lies in the fact that they are naturally occurring estrogens [6] affecting humans and several animal species. Swine is the mostly affected species by ZEA. It is known than ZEA causes hormonal disorders and is associated with human breast cancer [1,6]. Additionally, ZEA presence in feed during pregnancy may reduce the survival rate of the embryo [1]. Moreover, ZEA provokes vulvar dilatation and redness, decreases sperm quality, and rectal prolapse [1]. Zearalenone consumption by dairy cows can be excreted into milk [6].

Fumonisins (FUM) are produced mainly by *Fusarium* species such as *F. verticillioides, F. proliferatum*, and also *A. niger* [2,6] (Table 1). FUM are divided into groups: A, B, C, and P. From these groups, only six fumonisins have been recognized: two from group A (FA1, FA2) and four from group B (FB1, FB2, FB3, FB4) [6]. FB1, FB2, and FB3 can be found in nature, but only FB1 turns up in high percentage and constitutes the most toxic one. The FUM health effects on humans have not yet been investigated. On the other hand, surveys have shown that fumonisins cause serious damage in animal heath such as cancer, especially in the liver and kidney [2,6]. Additionally, FUM contamination causes an illness in horses called leukoencephalomalacia (ELEM) and a syndrome known as pulmonary edema and hydrothorax in pigs [1,2]. It is also known that FB’s cause damage on neural and liver tissues in fish [1].

Trichothecenes are a large family of chemically related mycotoxins produced by various fungal genera such as *Fusarium, Myrothecium, Trichoderma, Trichothecium, Cephalosporium, Verticimonosporium,* and *Stachybotrys* [2]. Trichothecenes are divided in four groups: A, B, C, and D. Most notably, group A included the most toxic compounds (T-2 and HT-2 toxins, diacetoxiscirpenol) and group B consisted of deoxynivalenol and nivalenol [6]. In addition, *F. langsethiae*, *F.poae, F. sporotrichioides, F equiseti,* and *F. acumninatum* are responsible for mycotoxins, which belong in group A [2]. Deoxynivalenol (DON) is produced by *F. graminearum, F. culmorum, F. cerealis (F. croockwellense), F. sporotrichioides, F. poae, F. tricinctum*, and *F. acuminatum* [2,6]. HT-2 and T-2 toxicity associated with growth retardation, myelotoxicity, hematotoxicity, and sepsis on contact sites [2]. DON intake causes damage in the digestive system and reduction in food appetite [6]. Nivalenol (NIV) is produced by *F. cerealis, F. crookwellense, F.poae, F. nivale, F. culmorum*, and *F. graminearum*. NIV intake causes serious damage on bone marrow, intestinal membranes, and lymphoid organs as well as erythropenia, leycopenia, hemorrhage, and diarrhea [6].

Sterigmatocystin (STC) is produced by more than 50 fungal species with the main source that of *Aspergillus* [7,8,9], notably *Aspergillus flavus, A. parasiticus, A. versicolor, A. nidulans,* and *A. versicolor* [7,10]. Sterigmatocystin has been accused of tumorigenicity, hepatocellular carcinomas, angiosarcomas in brown fat, lung adenomas, and hemangiosarcomas in the liver and pulmonary adenomas in both human and animals [7,11]. As far as animals are concerned, surveys have shown that STC is hepatoxic in both poultry and pigs, nephrotoxic in poultry, and extremely toxic in fishes. In cattle, bloody diarrhea, which can even lead to death, has been observed [7,11]. It is noteworthy that this mycotoxin group can be found not only in food and animal feedstuff, but also in indoor environments to wet building and housing materials [11,12].

Ergot alkaloids (EAs) have gained considerable attention with more than 50 compounds to have been identified including, but not limited to ergometrine, ergotamine, ergosine, ergocristine, ergocryptine, ergocornine, and the corresponding inine epimers [13,14,15]. EAs are produced by the *Claviceps* and *Epichloë* fungus [13,14,15]. Ergot alkaloids have been accused of neurotoxicity, weight reduction, and imbalance of the endocrine system (alter some hormones’ normal levels) [13,14].

Furthermore, Phomopsins are significant mycotoxins, although scarce data exist on their effects [16]. Phomopsins have been classified into five groups: A, B, C, D, and E, of which the most toxic is group A [16]. These mycotoxins are produced by *Diaporthe toxica* (*Phomopsis leptostromiformis*) [16,17]. Experimental results showed that these modified polypeptides are nephrotoxic, immunotoxic, hematotoxic, genotoxic, and carcinogenic [16]. Additionally, these mycotoxins are hematotoxic for animals and cause liver cancer in rats [16]. Phomopsin intake seems to be the cause of Lupinosis in shepherd animals [17].

Citrinin (CTN/CIT) is produced by three different genera of fungi: *Aspergillus, Penicillium*, and *Monascus* [9,12,18,19]. Several research data have described the toxicity of this secondary metabolite in the kidneys, liver, and the immune system [18,19,20]. Furthermore, citrinin may cause DNA damage and cancer [19]. It is noteworthy that this polyketide mycotoxin is detected in various herbal medicines [9,19].

Enormous interest exists in *Altenaria* mycotoxin research because of their frequent presence in food and feed [19,21]. *Altenaria* species are synthesized by *Altenaria* fungi and produce more than 70 toxins, but only a few have been featured as mycotoxins both for humans and animals [9,19,21]. Some complexes include, but are not limited to altenuene, tenuazonic acid, alternariol monomethyl ether, and alternariol [9,21]. Research results have shown that *Altenaria* compounds are phytotoxic, fetotoxic, genotoxic, and responsible for mutagenicity and teratogenicity in both humans and animals (especially mice, chicken, and dogs) [19,21,22].

Mycotoxin contamination of food has enormous economic and commercial implications [1,3,23,24,25]. Several times, the whole grain batch may need to be destroyed, more often maize, wheat, rye, barley, and oat [1]. Surveys have shown that economic losses can range from hundreds of million to billion dollars per year [25]. According to the FAO, financial damage amounts to twenty-five percent of the global harvest [3]. It is important to note, that these data were modified due to population growth from year to year, which causes an increase in production to meet mankind’s needs, so the level of mycotoxins increased due to the extension of storage time [24]. Loss incidents due to mycotoxins have been noticed in over a hundred countries [25].

Responsible fungal genera differ from country to country because of environmental conditions that favor species production [23]. The presence of mycotoxins reduces the crop quantity and quality as well as livestock production [3,23]. Moreover, economic losses can be indirect such as treatments that humans and animals may need after infected food consumption and reduction in the final product price due to the raw materials used [3,23]. Moreover, business related to crops may fail or lose profits [23]. An extra cost is that contaminated crops cannot be used as seeds the next year [23]. The most important commercial implications are losses of foreign exchange earnings due to mycotoxin contamination of food [23]. According to the literature, mycotoxins can be found in both human food and in animal feed. It is noticeable that the same fungal genera are responsible for the appearance of different mycotoxins. The results of the surveys are listed in Table 1.

Legislation and regulations on mycotoxins are continually evolving, since countries recognize that addressing mycotoxin contamination in feeds and foods will portray plenty of advantages such as decrease health-care expenses, improvement of the international trade, and promotion of sustainability [26]. Due to the aforementioned detrimental effects of mycotoxins, the European Union has set certain limits via regulations on their content in food and feed to preserve the health of both citizens and animals (EC, 1126/2007; EC, 165/2010; EC, 574/2011; EC, 594/2012; EC, 212/2014, in addition, the European Union provided recommendation EC, 2016/1319). The regulations and recommendations are presented in Table 2. Several other countries have set maximum mycotoxin limits. U.S. regulations concerning aflatoxin contaminated foods for humans are 10-fold higher than that of the EU, while those of India are 15-fold [26].

The detrimental effects of mycotoxins on livestock may result in morbidity and mortality [19,27]. Mycotoxins are capable of inducing several imbalances in the cellular level, resulting in acute or chronic health issues on animals. The induction of oxidative stress and generation of reactive oxygen species (ROS) may be a major trigger of detrimental outcomes [28]. Cells produce ROS and reactive nitrogen species (RNS) as a result of their physiological metabolism or as signaling molecules capable of regulating substantial homeostatic functions [29]. Another crucial role of ROS emerges through the phagocytic properties of immune cells [30]. It seems that there are two faces of ROS, redox signaling and oxidative stress, contributing to both physiological and pathological conditions. Within the range of low to moderate concentration levels, ROS regulate normal cell function, whilst at their highest levels, their biochemical instability damages cell component such as lipids, proteins, and DNA, resulting in oxidative stress [31]. However, the organism regulates an efficient network of mechanisms to counteract the detrimental effect of ROS, which includes enzymatic or endogenous and non-enzymatic (exogenous) factors [32].

Imbalance between free radicals generated by mycotoxins and the antioxidant defense system triggers a cascade of damaging effects [28]. More specifically, in vitro studies have reported that AFB1 induced downregulation of antioxidant enzymes such as superoxide dismutase (SOD), glutathione peroxidase (GSH-Px) and catalase, resulting in increased lipid peroxidation by-products (malondialdehyde; MDA) and a severe decrease in the levels of the predominant exogenous antioxidant compound, namely, reduced glutathione (GSH) [28]. Liver and kidneys are considered the main organs of the mycotoxins’ metabolism. Proper hepatic function is evaluated through several laboratory analyses including, but are not limited to, the determination of alanine aminotransferase (ALT) and aspartate aminotransferase (AST) levels. Increased ALT and/or AST activities are indicative of hepatocellular injury [33]. Plenty of factors predominantly affect the cellular function and homeostatic balance in animals affected by mycotoxins. These factors include, but are not limited to animal species, time of exposure, age, production stage, and toxin co-contamination (synergetic effect). The most determinant factors seem to be contamination level, type of mycotoxin, and time of exposure (acute or chronic). Under this context, it has been observed that the antioxidant defense of cows could be triggered under a short time of exposure to AFB1 (3–7 days), resulting in increased SOD activity as a response to oxidative challenge [34,35]. Moreover, long-term exposure of cows up to nine weeks to AFB1 manifested in low SOD activity, total antioxidant capacity, GSH-Px, and high levels of plasma MDA [36]. This study aims to bridge the detrimental effect of mycotoxin contamination in livestock with the antioxidant status of animal organisms, which is related to the products’ oxidative stability.

## 2. The Effect of Mycotoxins on the Antioxidant Status of Pigs and Poultry

Mycotoxins are considered one of the main contaminants in animal diets and their presence might damage livestock health [38,39]. The intensive rearing of poultry and swine might pose a risk for animal health and production because of the high consumption of cereals and oilseeds, which are more likely to contain mycotoxins [40,41,42]. Mycotoxins affect several organs such as the gastrointestinal system, liver, and immune system, and in general reduce productivity. Although one mycotoxin might be harmful for animals, the presence of more can be more toxic due to their synergism. Some of the most common species that can be found in feeds are AF, OTA, ZEA, FUM, DON, and T-2 toxin.

The toxic effect of mycotoxins can lead to oxidative stress (OS) and the generation of free radicals [43,44]. The increased number of free radicals in accordance with the malfunction of antioxidant system damages DNA, proteins, and lipids [45]. Oxygen free radicals and antioxidants are produced normally by cells in a balanced range. Exterior parameters can promote the generation of oxidative stress and an overproduction of free radicals [46], causing an imbalance in the homeostasis mechanism of the cells. Disruptions of the antioxidant system and excess generation of free radicals may lead to oxidative stress [47]. Valco et al. [48] stated that oxidative stress exists when the antioxidant capacity of a cell is overtaken due to the over production of reactive oxygen species (ROS), like the hydroxyl radical (HO^•^), perhydroxyl radical (HOO^•−^), superoxide anion (O_2_^•−^), and RNS including nitric oxide (NO). The excessive number of ROS species might cause an alteration or a generation of several intracellular mechanisms that oxidate DNA, proteins, and membrane lipids. Cell death is more likely if lipid peroxidation occurs, indicating the serious consequences of the toxicity of mycotoxins [49]. It is not clear if mycotoxins induce lipid peroxidation by triggering free radical production or by undermining the antioxidant defense. In order to tackle this situation, cells use primary and secondary enzymatic systems to avoid excessive damage [48]. Antioxidant enzymes such as superoxide dismutase (SOD), catalase (CAT), glutathione reductase (GR), and glutathione peroxidase (GSH-Px) compose a primary system to cope with free radicals or create a mechanism with glutathione (GSH).

Nutritional stress factors are responsible for negative effects in cell homeostasis. Mycotoxins are such kinds of factors and seem to have a negative impact on antioxidant enzyme function (Table 3). Galvano et al. [50] reported that AF are one of the most dangerous mycotoxin species and evaluated several dietary strategies to counteract the effects of mycotoxins. Alterations may occur depending on the mycotoxin species, the dose, and the duration of exposure, or in the presence of other antioxidants. An antioxidant enzyme may increase, if an oxidative stress occurs, or decrease, depending on the action of the mycotoxins.

Several studies have been conducted to examine the effects of several mycotoxin species on antioxidant enzymes in poultry and swine (Table 3). Results by Chen et al. [51] indicated the generation of oxidative stress in the spleen of chickens after the consumption of AFB1, as shown by the decreased levels of antioxidant enzymes such as GSH-Px, GR, CAT, and levels of malondialdehyde (MDA) and GSH. In agreement with the aforementioned study, Shahid et al. [52] found an increased rate in MDA, while total superoxide dismutase (T-SOD), GSH-Px, CAT, GR, and glutathione-S transferase (GSTs) were decreased in samples of liver and serum, as an effect of the contamination of one-day-old chick diets with a 1 mg AFB1/kg diet. These decreased activities of the above enzymes might lead to the generation of hydroxyl radicals, which play an important role in the lipid peroxidation process [53].

In another study [54], broilers that consumed a diet with 1 ppm aflatoxin B1 gained less weight, had lower feed intake, and at the same time, the activity of SOD was increased while that of CAT was decreased compared to the control group. MDA levels in serum were higher in broilers fed aflatoxin. Moreover, the control group had a lower activity of AST and ALT compared to the AFB1 group. Additionally, blood glucose was decreased and both cholesterol and triglycerides in the AFB1 group were increased. Similarly, Eraslan et al. [55] reported that exposure to AF at high doses caused lipid peroxidation in broilers. Li et al. [56] reported the effect of adding OTA in the diet of broilers in the inclusion rate of 50 μg/kg. MDA levels in kidneys were increased while the total antioxidant capacity (TAC) was decreased and levels of SOD, CAT, and GSH were markedly lower than in the control group. It was suggested that OTA induced the production of reactive oxygen, resulting in oxidative stress in the kidneys of chickens.

Yang et al. [33] carried out an in vivo and in vitro trial feeding broilers with a diet contaminated with T-2 and HT-2 mycotoxins. In the in vivo experiment, a reduction in the body weight and weight gain were observed, and the feed conversion ratio was worse compared to the control. These results were most prominent in the group with the highest concentration of toxins (4 mg/kg T-2 and 0.667 mg/kg HT-2). In the in vitro trial, a reduction of the GSH concentration in cells incubated with increasing concentrations of T-2 and HT-2 mycotoxins was reported (Table 3). Moreover, ALT/AST, GSH-Px, CAT, and SOD activities as well as MDA concentration were increased compared to the control. These results suggest that oxidative stress might be induced by the combination of these two toxins and is dose-related. Similar results were reported by Oskoueian et al. [57] after the in vitro application of AFB1 mycotoxin in the hepatocytes of five-week-old roosters. Antioxidant enzymes were negatively affected and at the same time, MDA levels increased. Similarly, Dvorska et al. [58] reported that the presence of the T-2 toxin in the broilers diet at 8.1 mg/kg fed for 3 weeks resulted in a decrease in the concentration of selenium, α-tocopherol, carotenoids, ascorbic acid, Se-dependent glutathione peroxidase (Se-GSH-Px), and reduced glutathione in the liver.

Studies have been conducted to determine the consequences of mycotoxins in the antioxidant status of pigs. Pigs are the most sensitive animals toward the products of aflatoxins, and damage in the liver and gut were observed after their consumption [59,60]. For instance, Thanh et al. [61] carried out an experiment with 6-kg weaned piglets that were fed diets contaminated with DON or/and ZEA. The control group contained 0.8 mg/kg DON and the contaminated diet contained 3.1 mg/kg of DON and 1.8 mg/kg of ZEA. The combination of DON-ZEA did not have any impact on the performance parameters for pigs, but induced oxidative stress. This was affirmed by the high level of MDA in the plasma and SOD in the liver. Antioxidant enzymes and GSH concentrations in plasma and liver were not affected. On the other hand, Sun et al. [62] studied the effect of naturally fed contaminated corn with aflatoxins (20 μg/kg) and FUM in pigs (6.02 ± 0.83 kg BW). Growing performance parameters were not significantly different between the control and the treatment group. MDA concentration was not affected by the presence of aflatoxin in the diet. Da Silva et al. [63] studied the intestinal explants of pigs after exposure to FB1 and/or DON in the treatments: DON 10 μM, FB1 70 μM, DON 10 μM + FB1 70 μM. From the results, it can be summarized that GSH was lower in treatments with one mycotoxin or a combination compared to the control group.

Reduced activity of the enzymes CAT, SOD, GSH-Px in plasma and organs was observed when weaned pigs were fed with 320 ppb of pure AFB1 [64]. The total antioxidant capacity also decreased as an effect of AFB1. Ren et al. [65] used porcine splenic lymphocytes and treated them with different concentrations of DON, ZEA, and their combination. SOD, CAT, GSH-Px, and GSH decreased when lymphocytes were exposed in DON or ZEA even in the lowest doses, when compared with the control group. In the group of DON and ZEA combination, antioxidant enzymes were lower than in the groups of DON or ZEA separately. In agreement with the previous studies, MDA increased in the exposed groups and was higher in the combination group.

Antioxidants of natural origin may protect against the toxic effects of mycotoxins by increasing the function of antioxidant enzymes [66] and the total antioxidant capacity in broilers against those contaminated with AFB1 feeds in broilers [54].

## 3. The Effect of Mycotoxins on the Antioxidant Status of Ruminants

Within the ruminant sector, feed contamination with mycotoxins results in crucial economic losses and food safety concerns. The economic impact of mycotoxins could either be directly through the rejection of the contaminated animal products occurring in reduced revenues, or indirectly via the animal’s long-term health exposure. Specifically, contaminated animals often showed severe immunosuppression, leading them to infection susceptibility or preventative vaccination failure. In this section, the aim was to expand on the current knowledge of the mycotoxin effects on ruminant health through examining the potential burden of immune and antioxidant systems.

The effects of mycotoxins in ruminants are not as severe as in monogastric animals, since rumen microbiome is able to metabolize and biotransform some toxins, however, without necessarily eliminating its whole compound load [67]. Hence, it could be hypothesized that ruminants are less susceptible to mycotoxins than monogastrics. However, it has also been mentioned that certain mycotoxins cause direct toxicity in rumen microbes, first and foremost to the cellulolytic [68]. More specifically, fusaric acid has been shown to exert an inhibitory effect against *Ruminococcus albus* and *Methanobrevibacter ruminantium*, predominant rumen microbes that contribute to cellulose degradation and hydrogen neutralization within the rumen, respectively [69]. Since ruminant physiology is strongly dependent on rumen microbiome and their dynamic biochemical procedures, the direct effect of mycotoxins on the rumen habitat activates a domino effect of physiological imbalances. Such imbalances have been previously summarized by Gallo et al. [70] where AFB1, DON, Gliotoxin, and Patulin negatively affected rumen dry matter and NDF digestibility. A severe decrease in rumen fiber digestion activity may enhance the utilization of high fermentable carbohydrates, which along with a suppression of rumen pH, results in MDA escalation and total antioxidant capacity inhibition in blood and tissues [71]. Furthermore, mycotoxin negatively affects the microbial protein synthesis within the rumen, resulting in a negative protein balance (NPB) [68]. During the demanding peripartum period, ruminants are able to catabolize their muscle tissue in order to be supplied with essential amino acids (AA) to fulfil their high protein demands [72]. Muscle hypercatabolism causes a significant increase in RNS production that in turn disrupts the oxidative equilibrium [73,74]. This unfavorable condition is further burdened with the presence of AFB1, since it has been observed that the inclusion of AFB1 in lactating cows significantly decreases the feed intake [70]. In addition, high genetic merit cows may be in a negative energy balance (NEB) during the prepartum period and early lactation with an inability to meet their high energy and nutrient requirements, leading to lipid mobilization and in turn, increased formation of beta-hydroxybutyrate (BHBA) and non-esterified fatty acids (NEFA). Indeed, the NEB induced by undernutrition in pregnant sheep results in increased levels of H_2_O_2_ and MDA, while CAT and SOD activities were observed suppressed in both maternal and fetal livers, indicating a severe oxidative stress [75]. Considering the aforementioned, mycotoxin contamination in high performance ruminants and during their transition period can further burden their cellular homeostasis. In addition to the important role of rumen in volatile fatty acid production, the liver has a critical role in the metabolism of glucose, lipid and nitrogen metabolism, ketogenesis, immune function, ammonia circulating, hormone catabolism, and vitamin and mineral metabolism. Proper liver function is reflected in the activity of several enzymes, most notably AST and ALT. Increased AST activity is linked to oxidative burst since the cell damage is related to free radical production [61].

AFB1, OTA, and ZEA are considered to be the predominant mycotoxins in agricultural products [76]. Huang et al. [77] tested the aforementioned mycotoxin contamination in the diet of dairy goats, reporting an intense oxidative burst (Table 4). The combination of 50 μg AFB1, 100 μg OTA, and 500 μg ZEA/kg dry matter intake (DMI) significantly increased the MDA serum concentration, decreased the total antioxidant capacity, and decreased the activities of SOD and GSH-Px [77]. These metabolic alterations portray an increase in ROS production and foremost in the superoxide anion and hydrogen peroxidase as precipitated by their corresponding neutralization mechanisms (SOD, GSH-Px). It could be hypothesized that the formation of the above unstable radicals oxidized the polyunsaturated fatty acids (PUFA) of the cells’ phospholipid membranes and MDA and other by-products were produced. On a cellular level, the activities of AST, ALT, alkaline phosphatase (ALP), and total bilirubin (TBIL) were increased, reflecting severe damage either on the hepatocytes or on the cell membranes’ permeability [78]. In addition to the detrimental role of ROS on cell membranes, findings on interleukin 6 (IL-6) concentration indicate further imbalances in the immune system. Specifically, the increase in IL-6 might be attributed to the regulatory effect of mitogen activate protein kinase (MAPK), which is triggered by ROS and promote signaling for pro-inflammatory response [79,80].

Both level and type of mycotoxin are substantial parameters of the severity of toxicity. For instance, the combination of 50 μg AFB1 and 100 μg OTA was more toxic than that of 50 μg AFB1 and 500 μg ZEA/kg DMI, despite the fact that there were higher concentration levels of ZEA compared to OTA [77]. In agreement, Mohamed et al. [81] fed sheep with diets that were contaminated with 50 μg AFB1 and 100 μg OTA/kg DMI during the peripartum period. A significant milestone in this study was related to the survival rates of lambs, which dropped from 100% to 50% when mycotoxins were added to the animals’ experimental diet. This observation may be correlated to the aforementioned reports of the neutralization role of rumen microbiome toward mycotoxins. Since the newborn’s stomachs are still not developed and the microbe’s colonization is still in progress, the animals are prone to the deleterious effect of toxins in the same extent as monogastrics. The response of oxidative indices and antioxidant enzyme activities in the aforementioned study by Mohamed et al. [81] were relatively comparable to a previous work by Huang et al. [77], indicating a pronounced negative effect of AFB1 and OTA on the oxidative balance in small ruminants. In this context, Wang et al. [82] investigated the effect of 100 μg AFB1/kg in 60 day-old lamb diets reporting no mortality, while body weight gain was decreased approximately to half. These alterations in productive features can be attributed to immense oxidative damage, as demonstrated by glutathione and glutathione dependent enzymes in the liver and duodenal mucosa of lambs. Specifically, aflatoxicosis decreased GSH concentration and GSTs and GR activities in both tissues, suggesting an inefficiency in the neutralized formed ROS and an incapability in detoxifying cells from xenobiotics (GSTs) [83]. A comparable experimental trial was conducted by Nayakwadi et al. [84] in goat kids (2–3 month-old) by contaminating diets with 10 or 20 ppm T-2 toxin. In the same way, the lipid peroxidation index in the liver, intestine, kidneys, and spleen were significantly increased by the addition of mycotoxins. Observing the results of these studies, discrepancies were revealed related to the activities of CAT and SOD, indicating that depending on stress impact, antioxidant enzymes are differentially modulated (Table 4). Different mycotoxins may create different stress factors. Nayakwadi et al. [84] reported a lipid peroxidation rate caused by the T-2 toxin [82], which was less pronounced compared to that of AFB1, ZEA, and OTA [85]. It is worth mentioning that ROS production at low levels or in short intervals may trigger an upregulation of antioxidant enzymes due to increased demands for detoxification [31]. However, oxidative stress is well-justified as a result of either the generation of a higher concentration of ROS or decreased production of antioxidants within the cells [86].

More recently, Wang et al. [87] provided cows with two levels of mycotoxin contaminated cottonseed (Level 1: AFB1 + 80.13 μg ZEA/daily/cow; Level 2: 40.16 μg AFB1 + 160.26 μg ZEA/daily/cow), exceeding the limits of the EU for an interval of 14 days. There were no significant alterations in the antioxidant enzymes and oxidative indices, except for a decrease in the gamma-glutamyl transpeptidase (GGT) within the lower level of contamination. GGT is considered an indicator of liver function in ruminants [88] and its modulation in serum portray that liver function was negatively affected by contaminated cottonseed, although it is unclear why the levels did not change significantly in the higher dosage. Authors have suggested that the lack of immense biochemical alterations were attributed to the short experimental period. Another study by Wang et al. [89] using the same experimental subjects, but this time with higher levels of AFB1 (20 or 40 μg AFB1/kg DMI; approximately 400 and 800 μg AFB1/day) for a shorter time schedule (seven days), reported that MDA concentrations in serum was significantly increased while SOD activity was suppressed in high contaminated levels. In conclusion, the results of these studies showed that AFB1 contamination in cow diets was able to induce an immediate oxidative imbalance, whilst higher contamination levels further portrayed significant importance relative to their effects.

Experimental studies by Xiong et al. [36,90] described an aflatoxicosis effect in late and early lactation stage of dairy cows (Table 4). The results of both trials were comparable, showing increased levels of MDA in serum while the activity of GSH-Px was suppressed. However, TAC, SOD activity, and the concentrations of IgA and IgG were further decreased even within the lower contamination level (20 μg AFB1/kg DMI) in the case of the long-term contamination experimental trial (nine weeks) in early lactating cows [36]. Discrepancies over time, like those reported, may be attributed to the accumulation of mycotoxins in the liver and kidneys of cows [91] compared to those contaminated for a shorter period of time. In addition, another substantial factor may be correlated to the imbalance of the antioxidant and immune systems, which are further burdened during the peripartum period; moreover, such toxicity finds the organism in a more unfavorable condition [90]. A more recent study by Elgioushy et al. [92] observed increased values of serum MDA and CAT activity while GSH-Px activity was decreased in cattle recharging a naturally contaminated diet with AFB1 (range from 5 to 20 ng/mL).

Another two in vivo studies in mid-lactating dairy cows were conducted by providing higher levels of aflatoxin (100 μg AFB1/kg DMI) in the TMR ration [34] or ingested through rumen canula [35]. In both studies, SOD concentration in serum was reported to be higher in aflatoxic animals compared to the control group ones, suggesting a response of cellular mechanism as a protection against the oxidative damage. On the other hand, in studies in dairy cows with lower levels of aflatoxins but for a longer contamination interval, SOD was found to be suppressed [36]. It seems that mycotoxin levels and duration of exposure may induce different effects on the antioxidant system. Specifically, it could be hypothesized that mycotoxin contamination induces a severe oxidative condition that in the first stage could trigger endogenous antioxidant mechanisms and increase enzyme activity, as observed by Weatherly et al. [34] and Sulzberger et al. [35], while longer contamination trials such as those by Xiong et al. [36] diminished the antioxidant defenses, resulting in the suppression of antioxidant enzymes.

Regarding the in vitro studies, a recent work by Pauletto et al. [93] on bovine hepatocytes reported a significant increase in MDA concentration in incubated cells with AFB1, while their transcriptional profile of antioxidant enzymes (CAT, SOD, GSH-Px) were not affected. Two comparable studies conducted in vitro in bovine mammary epithelia cells that were treated with DON (0.25 μg/mL) for 24 h showed an increase in the concentration of MDA while TAC and GSH portrayed a decrease [94,95]. More specifically, Wang et al. [94] reported a lower activity of the SOD enzyme and Zhang et al. [95] observed the same trend in transcript levels (SOD1 and SOD2). In both experimental trials, despite the negative oxidative status that was observed, immunomodulating genes related to pro-inflammatory responses were reported in higher expression levels, suggesting a cytokine storm. Finally, a study by Bernabucci et al. [96], which incubated the peripheral mononuclear blood cells in aflatoxins and fumonisin medium in cows, reported an increase in the levels of MDA concentration and downregulated the signaling levels of SOD and GPX1.

Taking into account the aforementioned reports, ruminants may be less susceptible to the negative effects of mycotoxins since death occurrence is rarely observed in adult animals, despite the exceeding levels administered in trials. On the other hand, the majority of the current knowledge supports the idea that severe oxidative stress is induced by mycotoxin contamination. These assumptions should aid us in reconsidering the “innocent” term relative to the minimal susceptibility of ruminants toward mycotoxins, since the toxicity is further transferred to humans through dairy products and meat consumption. Without overlooking the detrimental consequences of mycotoxin metabolites in animal foods, additional deleterious molecules may be present in the case of mycotoxin contaminated ruminants such as alkanes, MDA, and 4-hydroxy-2-(E)-Nonenal. More specifically, it has been confirmed that MDA can modify double-stranded DNA by the formation of amino-iminopropene crosslinks between the NH_2_ groups of a guanosine base and the NH_2_ group of the complementary cytosine base [97]. In addition, MDA also has carcinogenic properties, based on experimental studies on rats and mice [98]. Therefore, within Europe following Commission Regulation (EC) No. 165/2010, the industry should determine the milk for mycotoxin contamination. In future, corresponding rules and policies should be implemented for lipid peroxidation products given their well-documented disastrous consequences for consumer health.

## 4. Prevention Strategies and Detoxification Technologies for the Mitigation of Mycotoxins in Animal Diets

Diet contamination with mycotoxins is a global problem that leads to livestock illnesses, severe economic losses, and adverse human health effects. Apart from the fact that the peri-harvest strategies should be in agreement with the good agricultural practices, much attention has been paid to develop innovative detoxification methods during the recent decades. The efficiency of the above approaches generally depends on the initial contamination levels, the achieved inactivation rate, their regular application possibilities, their safety, and their cost [100,101,102].

### 4.1. Good Agricultural Practices

Plant selection or breeding programs for mycotoxin resistance, appropriate use of fungicides–insecticides, crop rotation, proper soil and irrigation management, transportation, and packaging are the most important preventive measures against the contamination of animal feedstuffs by mycotoxins [100,103]. Moreover, the selection of the optimal harvesting period and the avoidance of mechanical injury results in a reduction of fungal infection in the field and as a result, the mycotoxin levels are determined at low levels in the harvested crop [104]. Proper pest management and storage conditions (duration, temperature, humidity) and regular commodity inspection through an appropriate control strategy also minimizes the extent of contamination by mycotoxins. Insects and rodents could act as carriers of fungi spores leading to their excessive proliferation and spread [103,105] Rapid turnover of feed within the animal unit also reduces mycotoxin production, since less time is available for fungal growth and toxin production [100].

### 4.2. Physical Detoxification Techniques

In the case of moderate to light mycotoxin contamination, physical methods such as sorting, winnowing, washing, milling, and floating could contribute in reducing mycotoxin levels by removing the more heavily contaminated particles [106,107]. Furthermore, the subjection of crops to rapid drying immediately after harvesting significantly reduces their moisture level and intercepts fungal growth and proliferation [108]. Thermal treatment such as the high temperatures used in frying, roasting, toasting, and extrusion have promising effects on reducing the mycotoxin content of a feed [109]. Irradiation using medium or long wavelength UVA and UVB also remove mycotoxins without severe adverse effects on organoleptic properties, but the high cost of irradiation units and the safety concerns related with its application have prevented its regular use [110,111].

### 4.3. Chemical Detoxification Techniques

Acids, alkalis, organic acids, and oxidizing agents have already been used with the intention to modify the bioavailability of mycotoxins [112]. Reaction of mycotoxins with bases such as ammonia and sodium hydroxide, or ozone and hydrogen peroxide may also result in the structural changes of mycotoxins and lead to their transformation into other compounds, the toxicity of which should be assessed [107,109]. Parameters that should be taken into consideration before the application of a chemical detoxification method are their safety, cost, efficiency, and the extent to which the nutritional content or the organoleptic properties of the feed are negatively affected [100]. During the recent years, nanomaterials such as selenium-, zinc oxide-, or copper-nanoparticles have been used as mycotoxin binders, leading to their removal [113].

### 4.4. Biological Detoxification Techniques

Fungi causing mycoses can be separated into two major categories, namely primary and opportunistic pathogens [38]. Primary pathogens affect healthy organisms with competent immune systems, while opportunistic pathogens make use of a compromised immune system of the host [38]. Fungi can be transmitted vertically and horizontally into plants and crops. During the horizontal infection, fungal endophytes are contagiously spread through ascospores and this transmission can be inhibited by the application of certain fungicides. Vertical transmission of the endophytic hyphae into seeds and seedlings is associated with the transmission of the fungus from generation to generation and is also very important, since these hyphae cannot be controlled by fungicides, they are neither latent nor dormant, but physiologically active and comprise the reservoir from which infection and toxin biosynthesis are activated [114]. Biology-based methods are therefore developed and are generally considered as safe and efficient without negative implications on the sensory attributes of the treated material and on the environment. The strategies of using naturally existing microorganisms including bacteria and yeasts or bioactive materials such as enzymes or polypeptides that biodegrade mycotoxins and alleviate their toxic effects have gained ground during the recent decades. The first method consists of the development of nontoxigenic strains of fungi that preclude or decrease the growth of their closely related toxigenic strains through the principle of competitive exclusion [115]. These bio-control strains can be applied directly to soil, but the most effective way is by combining the desired strain with a carrier/substrate such as a small grain before planting that provides a competitive advantage against toxigenic fungi [116]. On the other hand, specific enzymes can also accelerate chemical reactions in an efficient way and biodegrade mycotoxins [101]. Parameters that affect the effectiveness of a biological detoxification method are the stability of these agents at a variety of external conditions, the ease of their production, the safety of the detoxification metabolites, and the economic feasibility of such methods [101].

### 4.5. Feed Additives

The adsorption and bio-inactivation of mycotoxins via ingested feed additives has been extensively studied in livestock. Several substances such as lucerne, zeolites, bentonite, and bleaching clays act as mycotoxin-binding agents and prevent intestinal adsorption of the toxin by the animal through its diet. In detail, the above additives form stable complexes with mycotoxins, resulting in a reduction of their bioavailability [50,117]. Their effectiveness is related to the structures of both the binders and the mycotoxins (charge distribution, polarity, pore size, surface area) [50]. Among the problems of this approach is the risk of decreasing dietary vitamins, amino acids, and mineral availability. In order to overcome these constraints, biomass that contains yeast, lactic acid bacteria, and conidia of *Aspergillus* is used as a second-generation binder by providing numerous potential sites for mycotoxin attachment and ensuring improved tolerance by the animals due to its nature [118]. Potential adsorbents should possess improved binding ability against a wide range of mycotoxins, high adsorption capability, and limited binding to nutrients [101].

Dietary supplementation with natural antioxidants significantly delays or inhibits feed oxidation and protects cellular membranes, proteins, lipids, and nucleic acids against the toxic effects of mycotoxins [119,120,121]. Many vitamins such as vitamins A, E, and C have the potential to act as free radical scavengers and alleviate the negative implications of oxidative stress. In brief, the antioxidant properties of vitamin A rely on the prevention of mutagenic epoxides from binding to DNA, the inhibition of toxic substances, and the increase in levels of antioxidant enzymes (GSH and GSH-Px) [121]. Vitamin C is a powerful antioxidant that acts as a scavenger of oxygen- and nitrogen-based free radicals contributing to a delay in the lipid peroxidation rate and prevention of the nitrozation of the target molecules and regulation of antioxidant enzymes [121]. Vitamin E is a potent chain-breaking antioxidant that is capable of scavenging ROS and terminating free-radical chain reactions [122].

Dietary inclusion of carotenoids (i.e., crocin) in the diet of mice restored normal levels of biochemical parameters in the liver and kidney that were deteriorated by mycotoxin patulin [123] and alleviated ZEN-induced toxicity [124]. In a study with mice, the ameliorative effect of curcumin on lipid peroxidation in the liver and kidney induced by aflatoxin were demonstrated [125]. Accordingly, in a study with pigs, phytic acid has been shown to exert beneficial effects on the small intestine (jejunum), alleviating the changes induced by the mycotoxins DON and FB1 and protecting cells against oxidative stress [63]. Finally, several minerals like zinc and selenium are capable of protecting against mycotoxins as shown in several studies [58,126,127]. Most notably, organic selenium and modified glucomannans exerted a protective effect against the antioxidant depletion of avian liver due to T-2 toxicity [58]. Zinc was able to reduce the cytotoxicity of OTA via inhibition of oxidative and DNA damage and via regulation of the expression of several zinc-associated genes [126].

## 5. The Biotransformation of Mycotoxins and Presence in Animal Products

The passage of mycotoxins or their metabolites into animal products through the contaminated diet is an issue of great importance for the consumers, but also the market. There is a variation in tissue deposition of the above toxins among farm animals that is attributed to differences in their absorption and metabolism. In general, the accumulation of mycotoxins and their metabolites in animal muscle tissues is low, often below detection limits due to their intense metabolism in the liver [128,129,130]. Blood, kidney, and liver contain higher levels of mycotoxins and their metabolites than muscles and adipose tissue. As a result, special attention should be given if these offal are consumed [129,131,132]. Human exposure to mycotoxins through the consumption of meat products could be a result of aging or other processes such as dry-curing and the application of mycotoxin contaminated spices (e.g., nutmeg, peppers, coriander, and paprika). Mold species belonging to the genus *Penicillium* and *Aspergillus* are usually isolated in cured, fermented, or ripened meats and contribute to the acquisition of the organoleptic properties of these products. On the other hand, development of toxigenic fungi poses a great hazard for human health related with mycotoxin synthesis on these substrates [133].

The carry-over of mycotoxins through egg consumption has also been examined. As shown, residues of aflatoxins and their metabolites were lower than the detection limits [134] or their determined levels were 5-fold lower (<1 μg/kg) than the maximum residue limits (MRL) set by the EU [135] in eggs produced by hens fed with diets contaminated with these mycotoxins. Aflatoxins were also detected in egg and chicken meat samples from Pakistan, but their levels were also lower than the above MRL; the highest concentrations of these mycotoxins were found in liver [136].

In general, multi-exposure of humans to mycotoxins via milk consumption is observed. AFM1 is the hydroxylated derivative of AFB1 and is the most usual mycotoxin determined in milk due to its resistance in heat. Its permissive levels are 0.05 μg/kg milk in EU and is related to carcinogenic and mutagenic properties. Apart from AFM1, aflatoxins M2, B1, B2, G1, G2, OTA, FB1, ZEA, or their metabolites are also found in milk samples. Although several factors affect mycotoxin biotransformation in milk such as their molecular weight and lipophilicity, diet (forage–concentrate ratio), feed intake, digestion rate, animal health and productivity, season, and environmental conditions, the carry-over of the majority of them is limited and does not negatively affect human health according to the literature [137,138]. As stated previously, rumen plays an important role as a barrier against various mycotoxins in milk-producing animals as a significant number of them are inactivated or metabolized into less toxic forms. However, some of them may pass the rumen unchanged or be converted into metabolites that retain toxicity (i.e., AFM1) and pose a risk for human health. During recent years, the co-existence of several mycotoxins in milk that could affect their toxicity due to additive or synergistic effects has also been examined [139]. At the same time, the carry-over of mycotoxins into milk is usually examined in healthy animals with an intact blood–milk barrier. However, various systemic diseases and mammary infections might alter the functionality of this barrier, and hence transmission rates may be higher in daily practice [140].

Lactation stage is a parameter that mainly appears to influence AFM1 levels in cow milk; samples from early lactation have 3–3.5-fold higher AFM1 content compared to that of late lactation [141]. This seasonal trend in the levels of mycotoxins in milk is possibly related with the prolonged storage required for cattle feeds at early lactation, providing favorable conditions for fungal growth [142]. AFM1 is mainly determined in the casein fraction of milk, resulting in 3-fold and 5-fold higher levels in soft and hard cheeses, respectively, compared to the milk from which they were produced [143]. On the other hand, fermentation during yoghurt production significantly decreased AFM1 levels as a result of low pH, the formation of organic acids, and the presence of *Lactobacillus* sp. [144].

The majority of the data that exist on the effects of the ingestion of mycotoxin contaminated diets on the quality characteristics of the derived products is for eggs. In poultry, turkeys and ducks are the most sensitive species to AF and when they are fed with AF contaminated diets, they produce small eggs of poor quality and pigmentation, possibly as an effect of fat deposition in the liver, which impairs lipid metabolism and pigment deposition in yolk [102,134]. At the same time, reduced values for shape index, color [145], shell thickness, and strength [135] were observed in laying hens fed aflatoxin contaminated diets. Egg weight, relative yolk weight, albumen height, and Haugh unit were also decreased in laying hens fed with AF and DON contaminated diets [146]. Feeding broilers with an OTA contaminated diet resulted in decreased dressing percentage, carcass fat content, and breast meat water holding capacity and increased liver relative weight and longer small intestine and caeca [147]. It can be concluded that animal product quality is of paramount importance, thus both prevention strategies and detoxification technologies should be implemented.

## 6. Conclusions

Mycotoxins, for decades, have been an important threat for the livestock and agriculture sector. The present study concluded that mycotoxin contamination induced severe oxidative stress responses on both monogastrics (poultry, swine) and ruminants (sheep, goats, cows), which may be related to the products’ oxidative stability and shelf life. In future, a more holistic approach should be implemented on the mycotoxin problem without focusing only on meeting the current legislation and regulations. Nowadays, the current health crisis, the COVID-19 pandemic, has changed our perspective on health issues to a more holistic point of view, espousing ever more the One Health Concept, recognizing the connection between people, animals, plants, and the environment.

## Figures and Tables

**Table 1:** Selected mycotoxins, producing fungal species, and contaminated food/feed.

Mycotoxin	Mycotoxin-Producing Fungi	Food/Feed Material	References
Aflatoxins	*Aspergillus flavus*	Maize, peanuts, wheat, rice, sorghum, pistachio, ground nuts, tree nuts (almonds, walnut, hazelnut, brazil nuts), cottonseed, spices (cumin, black pepper, chili pods/powder), dried fruits (figs, raising, currant, sultanas, plums, date, apricots), cereals, soybean, cocoa, milk, milk products, meat, feeds	[1,2,3,4,5,6,25]
	*Aspergillus parasiticus, Aspergillus ochraceus, Aspergillus carbonarius,*	Maize, peanuts, brazil nuts, cocoa	[3,5]
	*Aspergillus niger*		
Ochratoxins	*Aspergillus ochrareus*	Barley, wheat, maize, cereals, dried vine fruits, wine, grapes, coffee, cocoa, cheese, feeds	[1,2,5,6,25]
	*Aspergillus niger*	Coffee, grapes, maize	[5]
	*Aspergillus carbonarius*	Maize, cereals, dried vine fruits, wine, grapes, coffee, cocoa, cheese	[2,5]
	*Penicillium verrucosum*	Barley, wheat, cereals, dried vine fruits, wine, grapes, coffee, cocoa, cheese, feeds	[1,2,3,6]
	*Penicillium viridicatum*	cereals, dried vine fruits, wine, grapes, coffee, cocoa, cheese, feeds	[1,2,6,25]
Zearaleone	*Fusarium graminearum*	Wheat, maize, cereal product, barley, feeds	[1,2,6,25]
	*Fusarium culmorum, Fusarium equiseti, Fusarium cerealis, Fusarium verticillioides, Fusarium incarnatum*	Wheat, maize, cereal product, barley, rye	[2,6]
Fumonisins	*Fusarium verticillioides, Fusarium proliferatum*	Maize products, sorghum, asparagus, feeds	[1,2,25]
T-2 and HT-2	*Fusarium langsethiae, Fusarium sporotrichioides*	Maize, wheat, barley, oat, rye	[2,6]
Deoxynivalenol	*Fusarium graminearum, Fusarium culmorum, Fusarium cerealis, Fusarium sporotrichioides, Fusarium poae, Fusarium tricinctum, and Fusarium acuminatum*	Maize, wheat, barley, oat, cereal, cereal product, feeds	[1,2,6]
Nivalenol	*Fusarium crookwellense, Fusarium poae, Fusarium nivale, Fusarium culmorum, and Fusarium graminearum*	Maize, wheat, barley	[3,6]
Sterigmatocystin	*Aspergillus flavus, Aspergillus parasiticus, Aspergillus versicolor, Aspergillus nidulans and Aspergillus versicolor*	Wheat, oats, ryes, barley, buckwheat, grain-based products, breakfast cereals, cooking oils, sorghum, maize on the cob, maize-based thickeners, maize syrup, polenta, tacos, tinned sweet maize, popcorn and maize snacks, cheese, nuts (peanut, hazelnuts), coffee beans, fresh fruits and sterilized fruits (grapes, plums, apples, pears, bananas and oranges), fruit juices (apple juices, blackcurrant juice and cherry juice), green vegetables and canned vegetables, beer, spices, animal feed	[7,11,20,37]
Ergot alkaloids	*Claviceps purpurea, Claviceps fusiformis, Claviceps africana, Neotyphodium spp.,*	Rye, rye-containing commodities, wheat, triticale, barley, millet, oat, grains, grass	[2,14,24]
Alternaria	*Alternaria alternata, Alternaria tenuissima, Alternaria arborescens*	Grain based products, all cereal grains, fruit and fruit products, vegetables and vegetable products, oilseeds, beer, wine	[2,6,24]

**Table 2:** Focus on European legislation and major regulations and recommendations regarding the maximum levels of mycotoxins in foods and feeds.

Mycotoxin	Foodstuffs	Maximum Levels (μg/kg)			EU Commission Regulations or Recommendations
Aflatoxins		B1	B1, B2, G1, G2	M1	
	Cereal products	2	4	-	165/2010
	Maize and rice to be subjected to sorting or other physical treatment before human consumption or use as an ingredient in foodstuffs	5	10		165/2010
	Raw milk, heat-treated milk and milk for the manufacture of milk-based products	-	-	0.05	165/2010
	Feed raw materials (concerns feed with a moisture content of 12%)	0.02	-	-	574/2011
	Complementary and complete feeding stuffs, except: (A) compound feeds for dairy cattle and calves, dairy sheep and lambs, dairy goats and kids, piglets and young poultry, and (B) compound feeding stuffs for bovine animals (excluding dairy cattle and calves), sheep (excluding dairy sheep and lambs), goats (excluding dairy goats and goats) and pigs (excluding piglets) and poultry (except chickens) (concerns feed with a moisture content of 12%)	0.01	-	-	574/2011
	Compound feed for dairy cattle and calves, dairy sheep and lambs, piglets, dairy goats and kids and young poultry (concerns feed with a moisture content of 12%)	0.005	-	-	574/2011
	Compound feeding stuffs for bovine animals (excluding dairy cattle and calves), sheep (excluding dairy sheep and lambs), goats (excluding dairy goats and young goats) and pigs (excluding piglets) and poultry (excluding from young) (concerns feed with a moisture content of 12%)	0.02	-	-	574/2011
Ochratoxin A	Unprocessed cereals	5			594/2012
	Feed raw materials Cereal products (concerns feed with a moisture content of 12%)	0.25			2016/1319 *
	Complementary and complete feed for pigs (concerns feed with a moisture content of 12%)	0.05			2016/1319 *
	Complementary and complete feed for poultry (concerns feed with a moisture content of 12%)	0.1			2016/1319 *
	Complementary and complete feed for dog and cats	0.01			2016/1319 *
Zearalenone	Unprocessed cereals (not maize)	100			1126/2007
	Unprocessed maize with the exception of unprocessed maize intended to be processed by wet milling	350			1126/2007
	Feed raw materials Cereal products (concerns feed with a moisture content of 12%)	2			2016/1319 *
	Feed raw materials maize by-products (concerns feed with a moisture content of 12%)	3			2016/1319 *
	Compound feed for piglets, gilts (young sows), puppies, kittens, dogs and cats for reproduction (concerns feed with a moisture content of 12%)	0.1			2016/1319 *
	Compound feed adult dogs and cats other than for reproduction (concerns feed with a moisture content of 12%)	0.2			2016/1319 *
	Compound feed sows and fattening pigs (concerns feed with a moisture content of 12%)	0.25			2016/1319 *
	Compound feed calves, dairy cattle, sheep (including lamb) and goats (including kids) (concerns feed with a moisture content of 12%)	0.5			2016/1319 *
Fumonisins	Unprocessed maize, with the exception of unprocessed maize intended to be processed by wet milling	4000			1126/2007
	Raw materials: maize products (concerns feed with a moisture content of 12%)	60			2016/1319 *
	Compound feed for pigs, horses (*Equidae*), rabbits and pets (concerns feed with a moisture content of 12%)	5			2016/1319 *
	Compound feed for fish (concerns feed with a moisture content of 12%)	10			2016/1319 *
	Compound feed for poultry, calves (<4 months) and lambs and young goats (concerns feed with a moisture content of 12%)	20			2016/1319 *
	Complementary and complete feed for adult ruminants (>4 months) and mink animals (concerns feed with a moisture content of 12%)	50			2016/1319 *
T-2 and HT-2 toxin	Compound feed for cats	0.05			2016/1319 *
Deoxynivalenol	Feed materials, cereal products with the exception of maize (concerns feed with a moisture content of 12%) by-products	8			2016/1319 *
	Feed materials—Maize by-products (concerns feed with a moisture content of 12%)	12			2016/1319 *
	Compound feed for pigs (concerns feed with a moisture content of 12%)	0.9			2016/1319 *
	Compound feed for calves (<4 months), lambs, kids and dogs (concerns feed with a moisture content of 12%)	2			2016/1319 *
	Compound feed (concerns feed with a moisture content of 12%)	5			2016/1319 *
Citrinin	Food supplements based on rice fermented with red yeast *Monascus purpureus*	2000			212/2014

* denotes Commission Recommendation (EU).

**Table 3:** Selected studies presenting the effects of mycotoxins on poultry and swine’s oxidative indices and antioxidant enzymes.

Animal Species	Mycotoxin Tested	Levels	Oxidative Indices	Antioxidant Enzymes	Other Indices	References
Broilers	Aflatoxin B1	(1) 0.15 mg AFB1/kg (2) 0.3 mg AFB1/kg (3) 0.6 mg AFB1/kg	↑ MDA ↑ GSH	Spleen: ↓GSH-Px ↓GR ↓CAT		[51]
Broilers	Aflatoxin B1	1 mg AFB1/kg	↑ MDA	Liver and serum: ↓CAT ↓ GSH-Px ↓ T-SOD ↓ GR ↓ GSTs		[52]
Broilers	Aflatoxin B1	1 ppm	↑ MDA ↓ TAC	Serum: ↑ SOD ↓ CAT	↑ AST ↑ ALT ↓ Glucose ↑ Cholesterol ↑ Triglyceride	[54]
Broilers	Aflatoxin B1	(1) 0.05 mg/kg (2) 0.1 mg/kg (3) 0.5 mg/kg (4) 1.0 mg/kg	↑ MDA	↓ SOD ↓ CAT ↓ G6PD ↓ GSH-Px		[55]
Broilers	Ochratoxin	50 μg/kg OTA	Kidneys: ↑ MDA ↓ GSH ↓ TAC	↓ CAT ↓ SOD ↓ CAT (mRNA expression) ↓ SOD(mRNA expression) ↓ GSH-Px(mRNA expression)		[56]
Broilers (and broilers hepatocytes cells in vitro)	T-2 toxin HT-2 toxin	(1) 1 mg/kg T-2 + 0.167 mg/kg HT-2 (2) 2 mg/kg T-2 + 0.333 mg/kg HT-2 (3) 4 mg/kg T-2 + 0.667 mg/kg HT-2 Hepatocytes treated for 24 h with 10, 20, 50 and 100 nM of T-2 and HT-2 toxins	↑ MDA	(Relative mRNA expression of in vivo and in vitro trials) ↑ GSH-Px ↑ CAT ↑ SOD	↑ALT ↑AST	[33]
Broilers	T-2 toxin	8.1 mg/kg	↓ reduced glutathione	↓ Se-GSH-Px		[58]
Chicken (hepatocytes cells in vitro)	Aflatoxin B1	5 μM	↑ MDA	↓SOD ↓CAT ↓ GR	↑IL1β ↑NFkB ↑TNF-α	[57]
Pigs (weaned)	Deoxynivalenol Zearaleone	(1) 0.8 mg DON/kg (2) 3.1 mg DON/kg + 1.8 mg ZEA/kg	Plasma: ↑ MDA Liver and plasma: −GSH	↑ SOD in liver ↓ GPX2 gene expression in jejunum		[61]
Pigs	Aflatoxins	20 μg AF/kg	−MDA		↑TNF-α	[62]
Pigs	Fumonisin B1 Deoxynivalenol	(1) 10 μM DON (2) 70 μM FB1 (3) 10 μM DON + 70 μM FB1	↓ GSH ↑ MDA ↓ TAC (ABTS)			[63]
Pigs (weaned)	Aflatoxins	320 ppb pure AFB1	↓ TAC	Plasma and organs: ↓ CAT ↓ SOD ↓ GSH-Px		[64]
Pigs (porcine splenic lymphocytes cells in vitro)	Deoxynivalenol Zearaleone	(1) 0.06, 0.3, 1.5, and 7.5 μg/mL DON (2) 0.08, 0.4, 2, and 10 μg/mL ZEA (3) DON + ZEA at 0.06 and 0.08 μg/mL, 0.3 and 0.4 μg/mL, and 1.5 and 2 μg/mL respectively	↑ MDA ↓ GSH	↓ SOD ↓ CAT ↓ GSH-Px		[65]

↓ = significant decrease; ↑ = significant increase; − = no significant alternations.

**Table 4:** Selected studies presenting the effects of mycotoxins on the ruminants’ oxidative indices, antioxidant enzymes, and cellular function.

Animal Species	Mycotoxin Tested	Levels	Oxidative Indices	Antioxidant Enzymes	Other Indices	Notes	References
Dairy goats	Aflatoxin B1 (AFB1) Ochratoxin (OTA) Zearaleone (ZEA)	50 μg AFB1/kg DMI 50 μg AFB1 + 100 μg OTA/kg DMI 50 μg AFB1 + 500 μg ZEA/kg DMI 50 μg AFB1 + 100 μg OTA + 500 μg ZEA/kg DMI	↓ TAC ↑ MDA	↓ SOD ↓ GSH-PX	↑ ALT ↑ ALP ↑ TBIL ↑ IL-6 ↓ IgA	OTA + AFB1 more detrimental than ZEA + AFB1	[77]
Goats (kids)	T-2 toxin	10 and 20 ppm	↑ MDA (Lipid peroxidation)	Liver, Intestines, Kidneys: ↑ CAT ↑ SOD		2–3 months old	[84]
Sheep (Peripartum period)	Aflatoxin B1 (AFB1) Ochratoxin (OTA)	50 μg AFB1 + 100 μg OTA/kg DMI	↓ TAC ↑ MDA	↓ CAT ↓ SOD ↓ GSH-PX	↓ TP ↓ ALB ↓ Chol ↑ ALT ↑ AST ↑ Urea	Lambs’ mortality	[81]
Lambs	Aflatoxin B1 (AFB1)	100 μg AFB1/kg DMI	↓ GSH Liver ↓ GSH Duodenal	↓ GSTs Liver and Duodenal ↓ GR Liver and Duodenal		2 months old	[82]
Cows	Aflatoxin B1 (AFB1) Zearaleone (ZEA)	Level 1: 20.08 μg AFB1 + 80.13 μg ZEA/daily/cow Level 2: 40.16 μg AFB1 + 160.26 μg ZEA/daily/cow	-MDA	-GSH-PX -SOD	↓ GGT	-14 days interval -Late lactation	[87]
Cows	Aflatoxin B1 (AFB1)	20 or 40 μg AFB1/kg DMI (app. 20 kg DMI/day)	↑ TAC ↑ MDA (40 μg)	↓ SOD (40 μg)		-7 days contamination interval -Late lactation	[89]
Cows	Aflatoxin B1 (AFB1)	20 or 40 μg AFB1/kg DMI (app. 17 kg DMI/day)	↑ MDA	↓ GSH-Px		-7 days contamination interval -Late lactation	[90]
Cows	Aflatoxin B1 (AFB1)	20 μg AFB1/kg DMI (app. 24 kg DMI/day)	↓ TAC ↑ MDA	↓ SOD ↓ GSH-Px	↓ IgG ↓ IgA	-Early lactation -9 Weeks contamination interval	[36]
Cows	Aflatoxin B1 (AFB1)	5–20 ng/mL (TLC assay)	↑ MDA	↑ CAT ↓ GSH-Px	↓ Total protein ↑ ALT ↑ AST ↑ ALP ↑ Creatine	Naturally contaminated feeds	[92]
Cows	Aflatoxin B1 (AFB1)	100 μg AFB1/kg DMI (21.9–23.4 kg DMI/day)		↑ SOD	↑ Glucose	-7 days contamination interval -Mid-lactation	[34]
Cows	Aflatoxin B1 (AFB1)	100 μg AFB1/kg DMI (21.4–22.8 kg DMI/day)		↑ SOD		-3 days contamination interval -Mid-lactation -ingested through rumen canula	[35]
Cows	Aflatoxin B1 (AFB1)	100 μg AFB1/kg DMI (24.9 kg DMI/day)		-SOD -GPX ↑ GPX1 (1)	-Chol -Albumin -BUN ↑ NFkB (1)	-Gene expression in Liver -Mid-late lactation -3 days contamination interval	[99]
Cows (bovine fetal hepatocytes cells in vitro)	Aflatoxin B1 (AFB1)	3.6 μM AFB1 in 6 × 103 hepatocytes	↑ MDA	-GSH-Px -CAT -SOD		transcriptional profiles using RNA -seq	[93]
Cows (bovine mammary epithelia cells in vitro)	Deoxynivalenol (DON)	Cells were treated with DON (0.25 μg/mL) for 24 h	↓ TAC ↑ MDA ↓ GSH	↓ SOD	↑ NFkB ↑ MyD88 ↑ TNF-α ↑ IL-1b ↑ IL-6 ↑ IL-8	-Higher cells’ apoptotic rate	[94]
Cows (bovine mammary epithelia cells in vitro)	Deoxynivalenol (DON)	Cells were treated with DON (0.25 μg/mL) for 24 h	↓ TAC ↑ MDA ↓ GSH	↓ SOD1 (expres.) ↓ SOD2 (expres.)	↑ NFkB ↑ COX-2 ↑ iNOS ↑ IL-1b ↑ IL-6 ↑ IL-8 ↑ TNF-α (Protein)	-Incubated for 9 h. -Decreased cell viability and proliferation	[95]
Cows in vitro (Peripheral blood mononuclear cells)	Aflatoxin B1 (AFB1) Fumonisin B1 (FB1)	0, 5, 20 μg/mL AFB1 0, 35, 70 μg/mL FB1	↑ MDA	↓ SOD (expres.) ↓ GPX1 (expres.) AFB1 5 μg ↓ GPX1 (expres.) FB1		2- and 7-days incubation	[96]

↓ = significant decrease; ↑ = significant increase; − = no significant alternations.
